# Does Valproic Acid Have Potential in the Treatment of Diabetes Mellitus?

**DOI:** 10.3389/fendo.2017.00147

**Published:** 2017-06-26

**Authors:** Aleksei Rakitin

**Affiliations:** ^1^Department of Neurology and Neurosurgery, University of Tartu, Tartu, Estonia

**Keywords:** valproic acid, glucose, insulin, histone deacetylase inhibitor, insulin resistance, valproate, diabetes, metabolic syndrome

Valproic acid (VPA), also known as valproate, is one of the most frequently prescribed antiepileptic drugs (1). An estimated more than one million people worldwide take VPA each day (2). VPA was first synthesized in 1882 by Beverly S. Burton as an analog of valeric acid, which is naturally produced by *Valeriana officinalis* (3). VPA is a branched fatty acid with eight carbons and has the simplest molecular formula among all anticonvulsants. Owing to its striking similarity to γ-aminobutyric acid (GABA), VPA has strong GABA-ergic effects (Figure 1). VPA was used initially as a solvent for organic compounds until its antiseizure activity was serendipitously discovered by Pierre Eymard in 1962 (4). After being approved for use as an anticonvulsant in France in 1967, VPA was marketed in more than 100 countries for epilepsy treatment (5). Since then, VPA has been successfully used for other indications, including bipolar disorder, migraine headache, and diabetic neuropathy-related pain (6, 7). After discovery of the histone deacetylase (HDAC)-inhibiting property of VPA, the drug was found to be effective in the treatment of leukemia and some solid tumors (8, 9).

**Figure 1:**
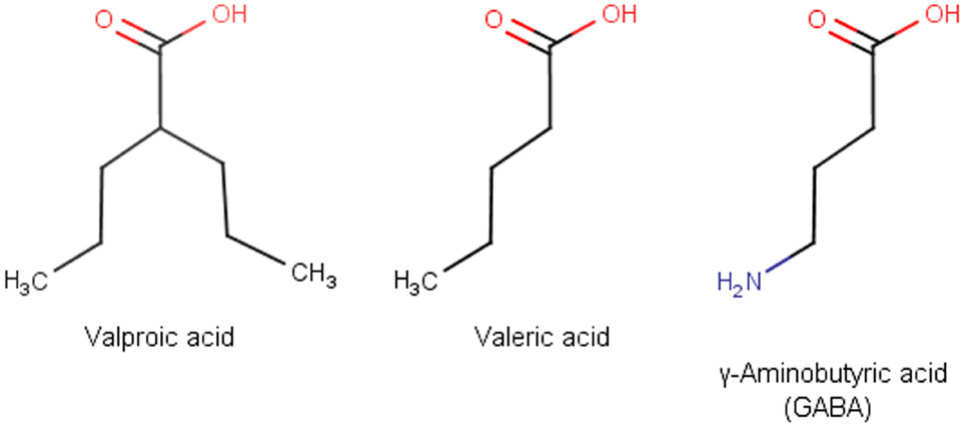
Structure of valproic, valeric, and γ-aminobutyric acid.

Weight gain is a frequently reported side effect of VPA (10). To date, however, only one double-blind prospective controlled study has specifically focused on this side effect. Patients treated with VPA for 32 weeks gained more weight than patients treated with the anticonvulsant lamotrigine (11). Results from other studies on this topic have been ambiguous, with several studies confirming weight gain in VPA-treated patients (12–14) but others failing to show this association (15–17). As a result, the true incidence and magnitude of this problem are not known (18). Few studies, with conflicting results, have examined the presence of metabolic syndrome (MS) in VPA-treated patients. Some of these studies focused only on specific populations, such as females (19) or children (20, 21), whereas others studied different metabolic side effects of VPA without direct assessment of MS occurrence (15, 22). Verrotti et al. showed that 40% of patients who gained weight during VPA therapy developed MS. However, this study lacked a control group of patients not taking VPA, and this result was similar to those reported in other studies carried out in obese patients who were not treated with VPA (21). Other researchers reported similar frequencies of MS between VPA-treated adults and control subjects (15, 23, 24). A study evaluating the prevalence of MS among Chinese adult obese patients with epilepsy treated with VPA showed a tendency toward a higher risk of MS compared to obese control subjects, without reaching the level of statistical significance (25). On the other hand, Kim and Lee showed a clear association between VPA monotherapy and MS in females with epilepsy (19). In a recent study from India, use of VPA was associated with a significant risk of MS in patients with epilepsy who were attending outpatient clinics (26). Given the diversity of results, further studies are needed to clarify whether VPA treatment is associated with a higher risk of developing MS.

Several attempts have been made to understand the metabolic effects of VPA. Some studies reported that patients on VPA treatment had increased homeostatic model assessment-insulin resistance (HOMA-IR) index values. As HOMA-IR is widely used for assessing insulin resistance (IR), researchers initially concluded that VPA caused IR (10). Later studies showed that the increased HOMA-IR values most likely reflected hyperinsulinemia, which can occur in VPA-treated patients. Increased insulin levels are found in both obese and lean patients treated with VPA, suggesting that hyperinsulinemia is not a consequence of increased weight but probably a direct effect of VPA treatment (27). The cause of hyperinsulinemia in these patients is not known, although several hypotheses have been proposed. For instance, VPA could interfere with insulin degradation during first passage in the liver, resulting in a higher peripheral concentration of insulin (27). Alternatively, VPA could directly stimulate pancreatic insulin secretion due to its GABA-ergic property (28). Patients on VPA treatment have been shown to have lower fasting plasma glucose concentrations, a finding that refutes the possibility of reduced insulin sensitivity (24, 27, 29). Turnbull et al. demonstrated a moderate fall in blood glucose levels after oral or intraperitoneal administration of VPA in Wistar rats (30), an effect that was confirmed in subsequent preclinical studies (31–33). VPA was effective in lowering blood glucose levels by potentiating insulin action in streptozocin-induced type 1 diabetic mice (34). Several clinical studies reported lower blood glucose levels in VPA-treated patients compared to controls (27, 29, 35, 36). Although hyperinsulinemia may be one cause of the lower blood glucose concentration, in some patients the lower blood glucose level was not associated with insulin concentration, suggesting the possible role of an insulin-independent mechanism (29, 36).

Valproic acid is a potent inhibitor of HDAC enzymes. HDAC inhibition promotes histone acetylation, leading to the relaxation of chromatin and facilitation of transcriptional activation. Recent findings highlight the crucial role of histone acetylation in the pathogenesis of type 2 diabetes mellitus (T2DM). Regulation of HDAC activity is a new approach to modify metabolism of glucose and fatty acid in the treatment of T2DM (37). HDAC inhibition can promote the development, proliferation, differentiation, and function of pancreatic β cells and ameliorate microvascular complications in later stages of disease (38). A recent study found that VPA attenuated diabetes-induced renal injury in a rat model of diabetic nephropathy by inhibiting the endoplasmic reticulum stress response. This effect may be due to regulation of histone H4 acetylation in promoters of the endoplasmic reticulum stress-associated proteins GRP78 and CHOP (39). Another study demonstrated beneficial roles of VPA in reducing fat accumulation, IR, and gluconeogenesis in a T2DM rat model. The antidiabetic effect of VPA was mediated through inhibition of forkhead box protein O1-mediated gluconeogenesis and promotion of glucagon-like peptide-1 action, probably through HDAC inhibition and associated mechanisms (32). A glucose-lowering effect was reported directly after acute intravenous administration of VPA during oral glucose tolerance test in patients with newly diagnosed epilepsy. This effect is probably too rapid to be mediated *via* HDAC inhibition and likely involves another mechanism (40). VPA has been successfully used for years in T2DM patients to treat neuropathic pain and has been suggested for use to treat diabetic polyneuropathy (6).

Valproic acid was introduced into clinical practice 50 years ago as a result of fortuitous chance. Today, VPA is used to treat many chronic diseases, and its tolerability and utility across different disorders are well established. Data from several preclinical and clinical trials suggest that VPA could have antidiabetic properties, mediated through yet-unknown molecular mechanisms. In addition to the development of new antidiabetic substances with HDAC-inhibiting properties, well-designed randomized control trials are needed to confirm the utility of VPA as a supplemental therapy for diabetes. Data on pharmacokinetics, oral bioavailability, half-life, and frequent side effects of this drug during long-term treatment are already available. For example, gastrointestinal side effects (e.g., nausea, vomiting, and gastrointestinal distress) occur in up to 25% of patients who take VPA and could restrict the potential use of this drug in patients with diabetes (41). Manifestations of gastric intolerance are less pronounced when the drug is an enteric-coated formulation or administered with food (1). Among the other relevant side effects of VPA are tremor, hair loss, thrombocytopenia, infertility, and teratogenicity. These side effects should be managed on a patient-by-patient basis, as is done now in patients with epilepsy, migraine, or bipolar disorder who are taking VPA. Females with childbearing potential should not use VPA (1). Unfortunately, because VPA is currently off-patent, there could be little economic interest for the pharmaceutical industry to conduct such clinical trials. Still, if the antidiabetic effect of VPA was to be confirmed, a subset of people with diabetes would benefit from having an additional inexpensive option to treat their condition.

## Author Contributions

Conception of idea and writing the opinion by AR.

## Conflict of Interest Statement

The author declares that the research was conducted in the absence of any commercial or financial relationships that could be construed as a potential conflict of interest.
